# Supplementary material for: A Portable Servoregulation Controller to Automate CO2 Removal in Artificial Lungs
Source: Bioengineering (Basel). 2022 Oct 21;9(10):593. doi: 10.3390/bioengineering9100593 (PMC9598878; doi:10.3390/bioengineering9100593)
Supplement: Supplementary file 1 [file bioengineering-09-00593-s001.zip › bioengineering-1921408-Supplementary.pdf]

Supplementary Materials: A Portable Servoregulation Controller to Automate CO_2 Removal in Artificial Lungs

Navid Shaikh, Andrew Zhang, Jesse Jenter, Brandon Nikpreljevic, John Toomasian, William Lynch, Alvaro Rojas-Peña, Robert H. Bartlett, and Joseph A. Potkay

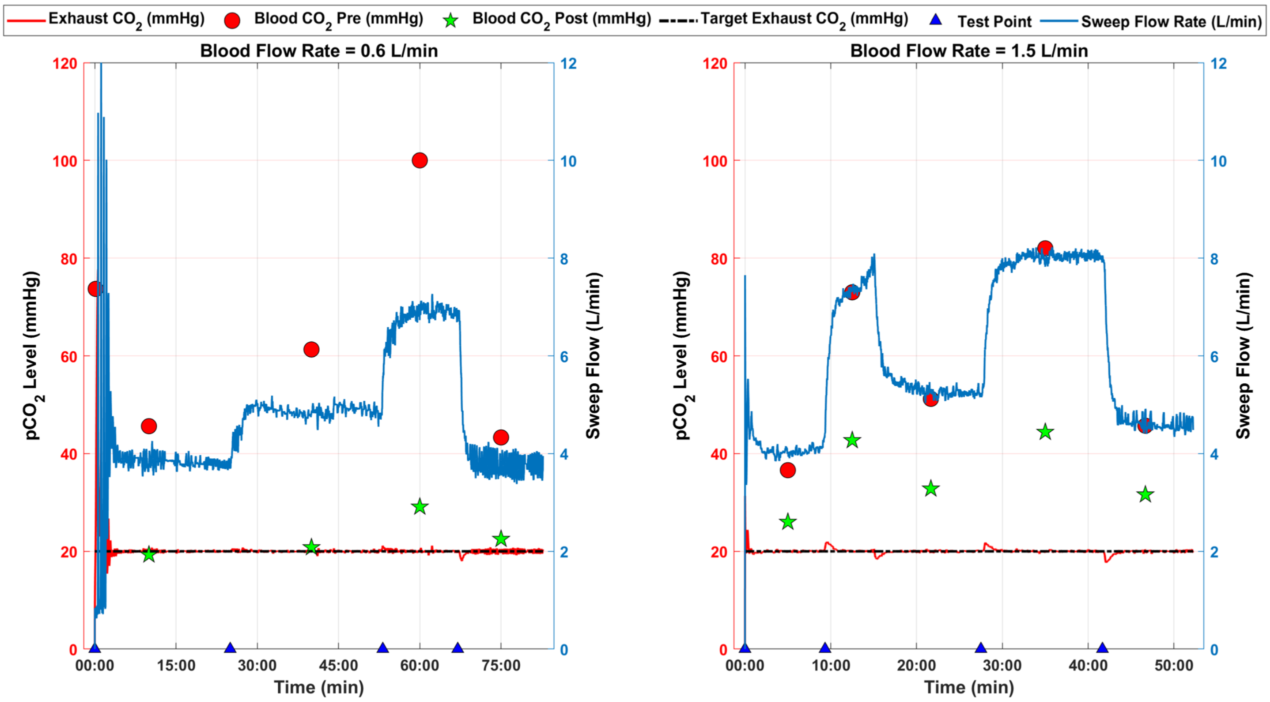

Figure S1. Data from *in vitro* studies at Artificial Lung(AL) blood flow rate of 0.6 L/min and 1.5 L/min. The target exhaust CO_2 level was maintained at 20 mmHg. The blue arrows represent the time points when the inlet blood pCO_2 levels were changed to challenge the tuning of the PID controller.

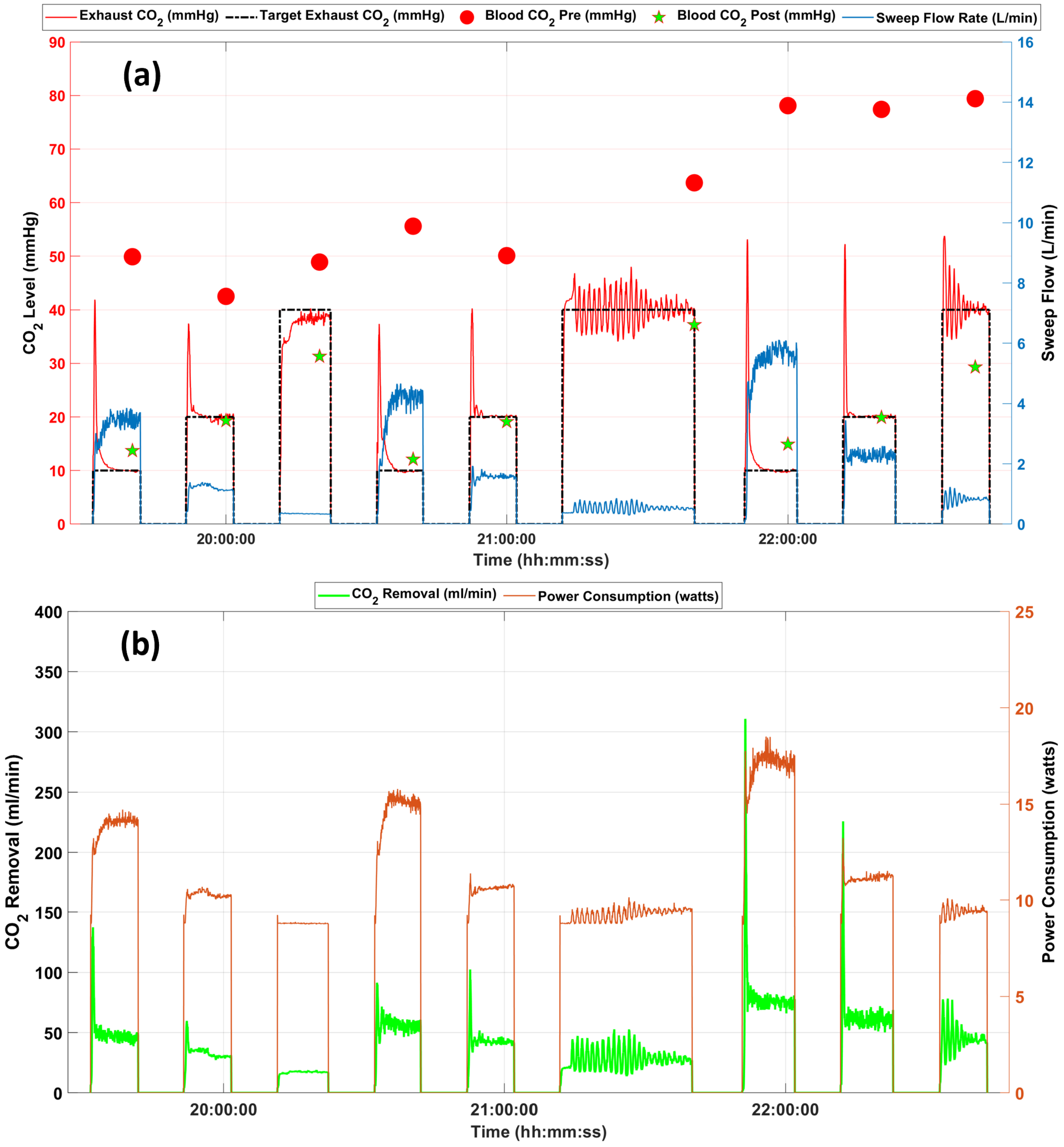

Figure S2. Raw data from *in vitro* studies at AL blood flow rate of 0.5 L/min showing (**a**) exhaust CO_2 levels, target exhaust CO_2 levels, sweep flow rates. (**b**) instantaneous CO_2 removal rate and system power consumption.

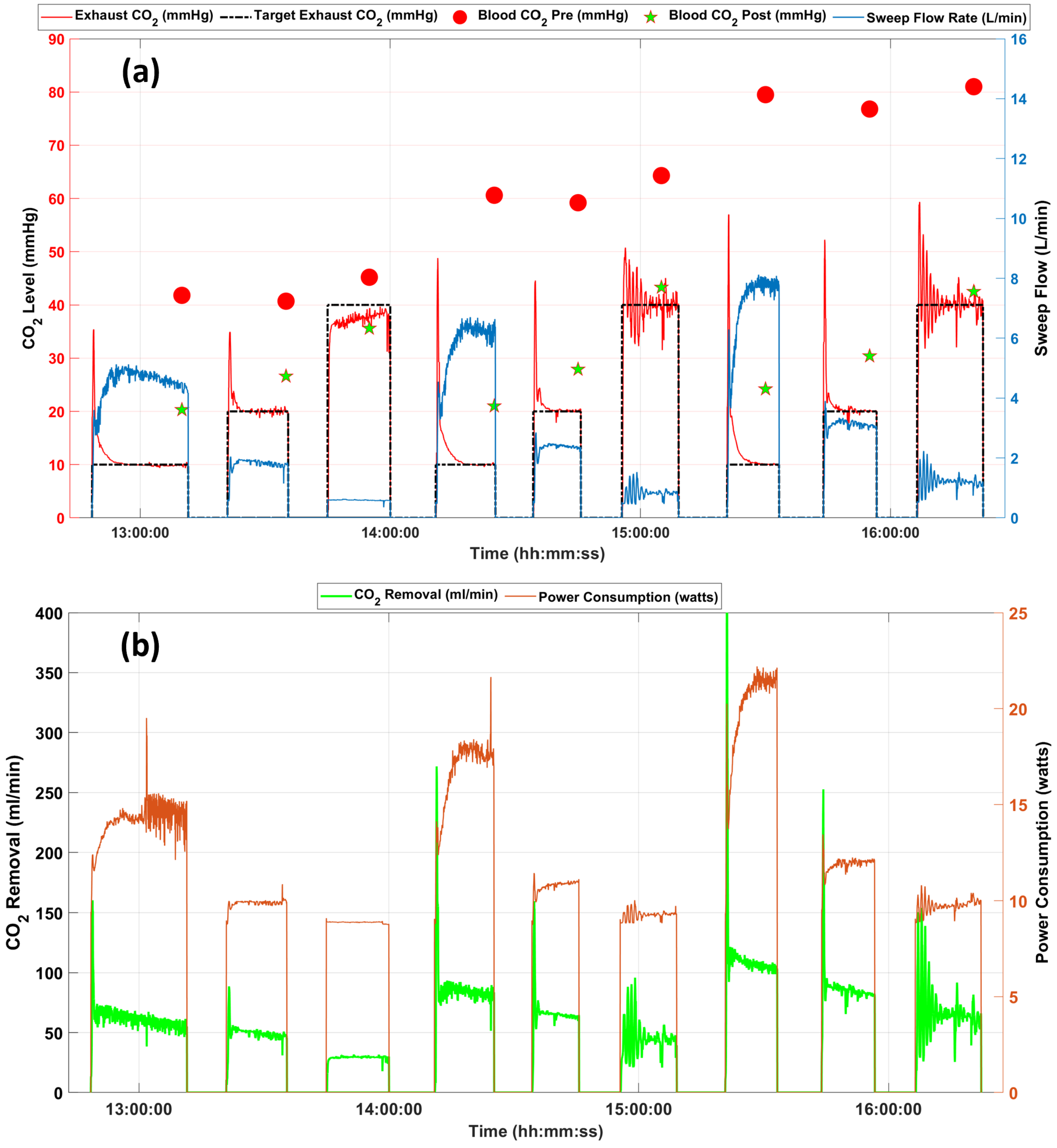

Figure S3. Raw data from *in vitro* studies at AL blood flow rate of 1.0 L/min showing (**a**) exhaust CO_2 levels, target exhaust CO_2 levels, sweep flow rates. (**b**) instantaneous CO_2 removal rate and system power consumption.

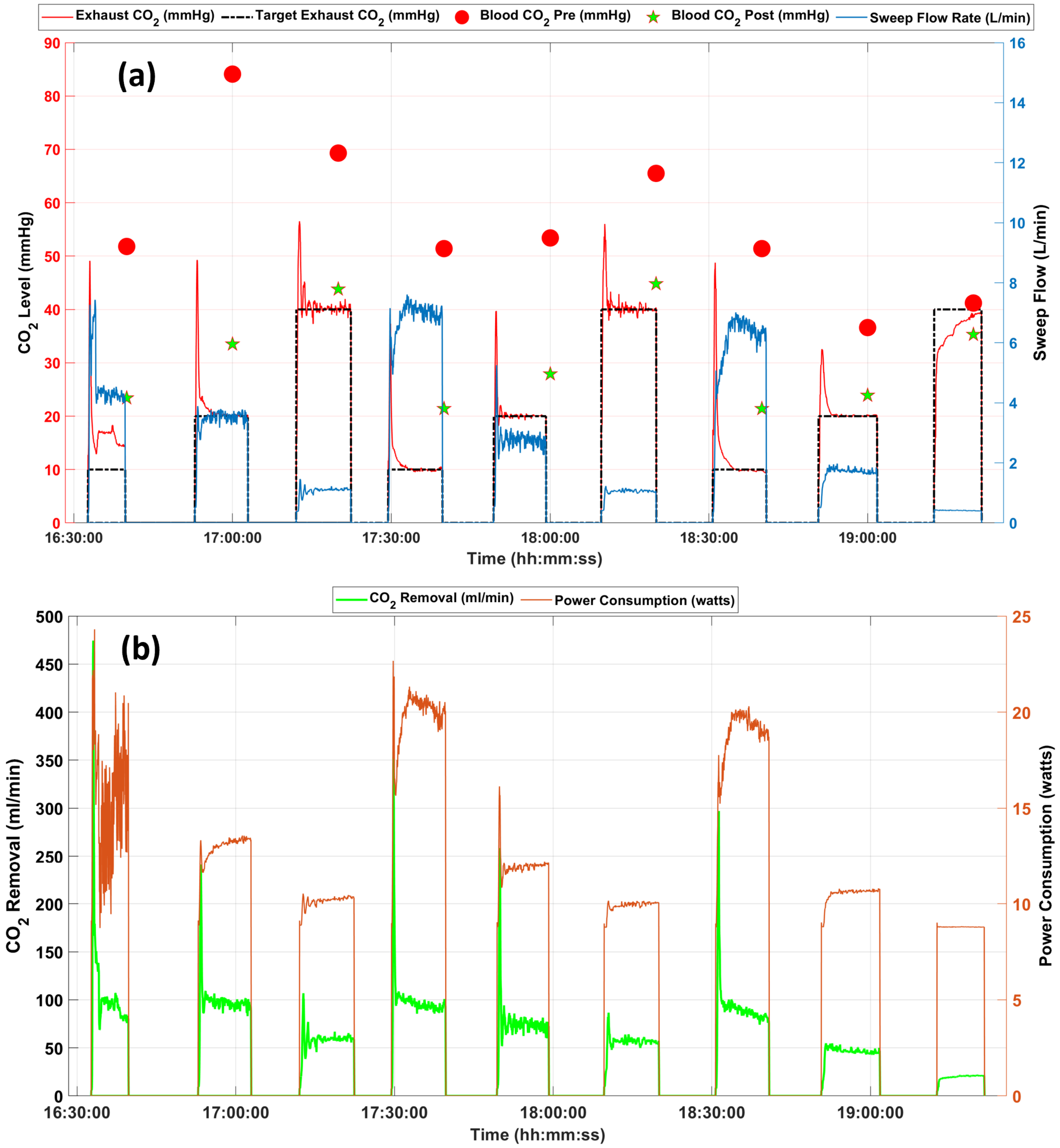

Figure S4. Raw data from *in vitro* studies at AL blood flow rate of 1.5 L/min showing (**a**) exhaust CO_2 levels, target exhaust CO_2 levels, sweep flow rates. (**b**) instantaneous CO_2 removal rate and system power consumption.
